# Supplementary material for: In-Situ Surface Modification of ITO Substrate via Bio-Inspired Mussel Chemistry for Organic Memory Devices
Source: Biomimetics (Basel). 2022 Dec 12;7(4):237. doi: 10.3390/biomimetics7040237 (PMC9775351; doi:10.3390/biomimetics7040237)
Supplement: Supplementary file 1 [file biomimetics-07-00237-s001.zip › biomimetics-2062802-supplementary.pdf]

Supporting Information

In-Situ Surface Modification of ITO Substrate via Bio-Inspired Mussel Chemistry for Organic Memory Devices

Minglei Gong[1,2], Wei Li[1], Fei Fan[2*], Yu Chen[1] and Bin Zhang[1*]

[1]Key Laboratory for Advanced Materials and Joint International Research Laboratory of Precision Chemistry and Molecular Engineering, School of Chemistry and Molecular Engineering, East China University of Science and Technology, Shanghai, China

[2]Shanghai i-Reader Biotech Co., Ltd. Shanghai, China

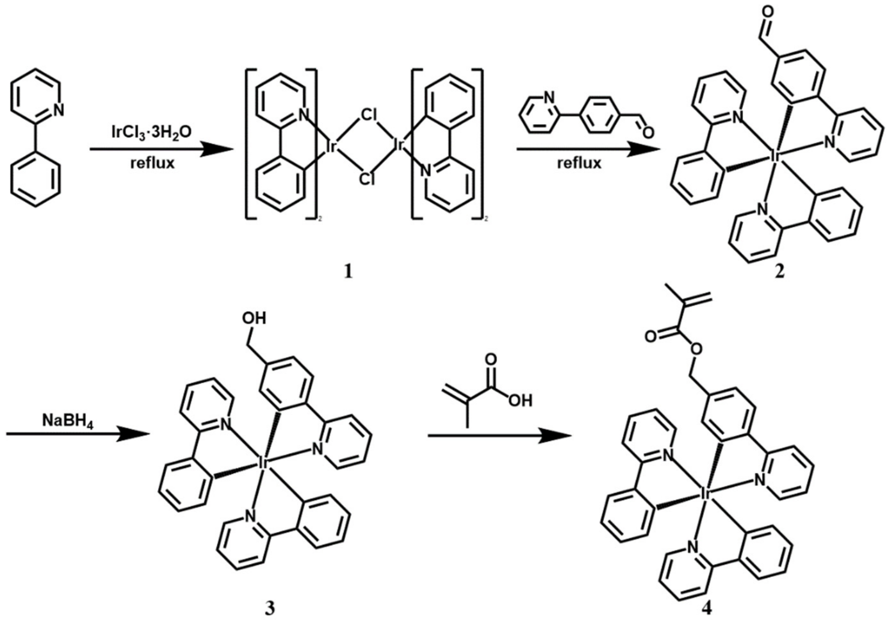

Figure S1. Synthesis of the iridium complex.

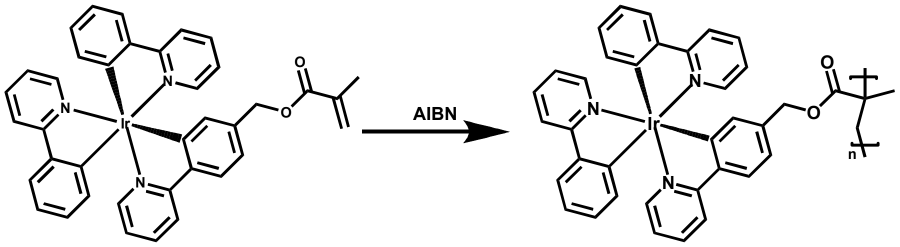

Figure S2. Synthesis route of the iridium polymer.

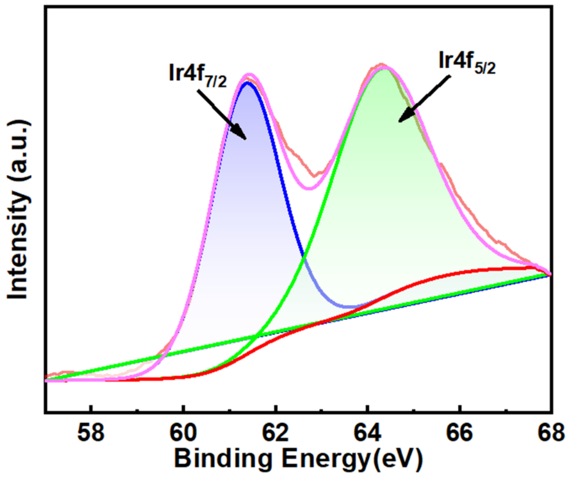

Figure S3. XPS Ir 4f core-level spectra of the iridium polymer.

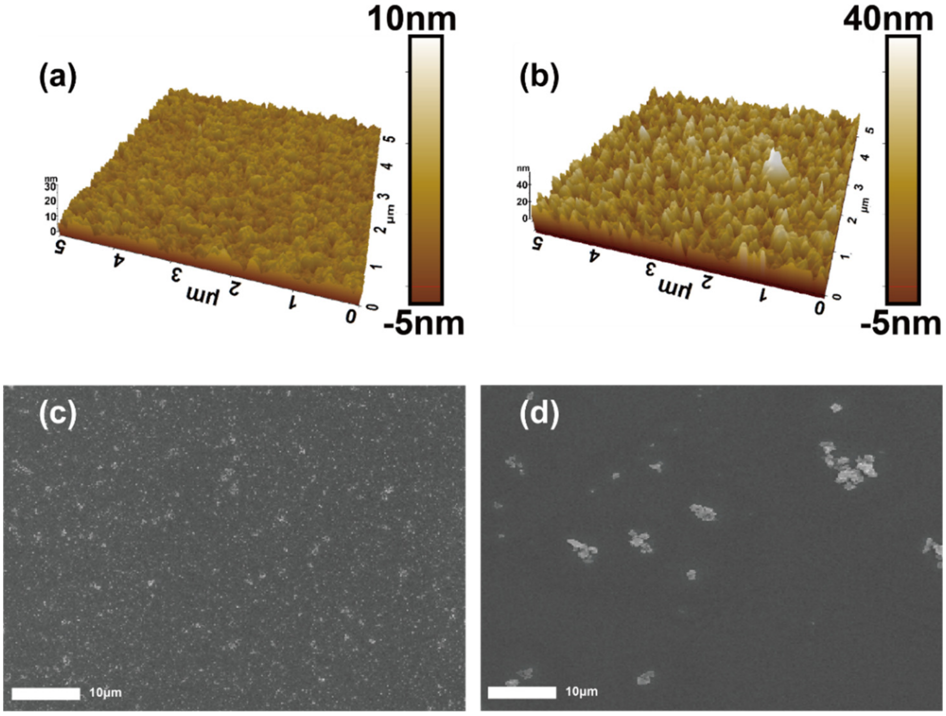

Figure S4. 3D AFM images of (a) PDA-PPy_3Ir/ITO substrate; (b) Ir complex spin-coated PDA/ITO substrate; (c) FESEM image of PDA-PPy_3Ir/ITO substrate; (d) Ir complex spin-coated PDA/ITO substrate.

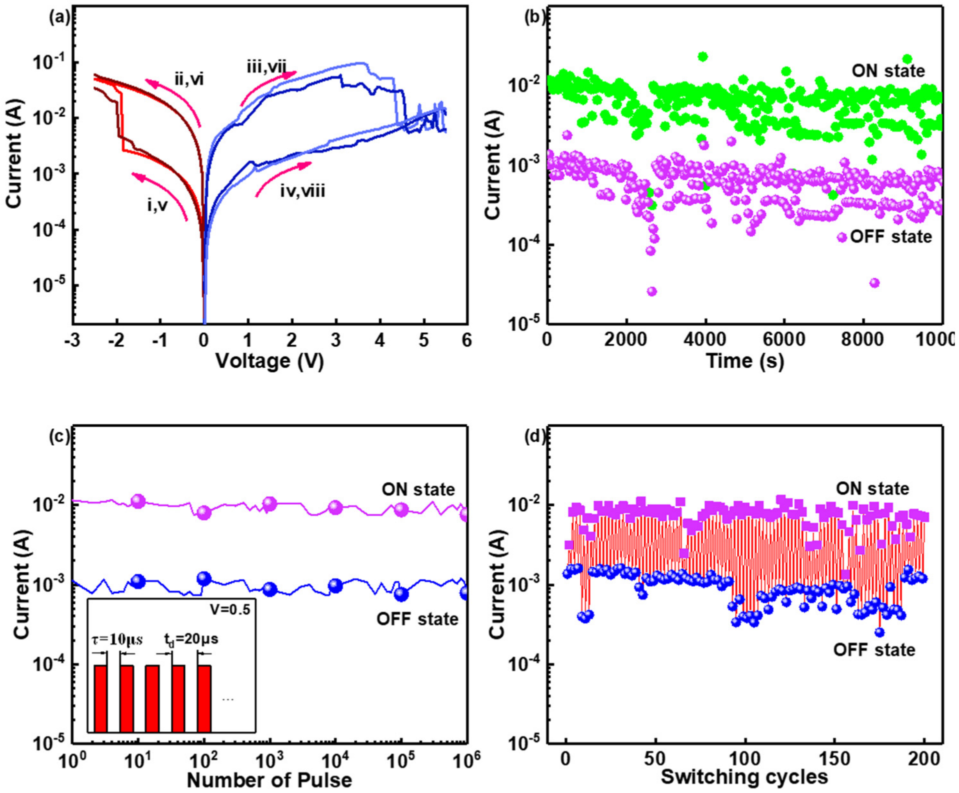

Figure S5. (a) *I-V* curves of Al/Ir polymer/PDA/ITO device; (b) stability of the device in the ON and OFF states under a constant voltage of -0.5 V; (c) effect of continuous read pulses of -0.5 V (pulse width = 10 μs, pulse period = 20 μs) in the ON and OFF states of the device; (d) endurance performances of the device (read at −0.5 V, with the switched pulses of −3 V and 5 V, pulse width: 100 ms; pulse period: 200 ms).

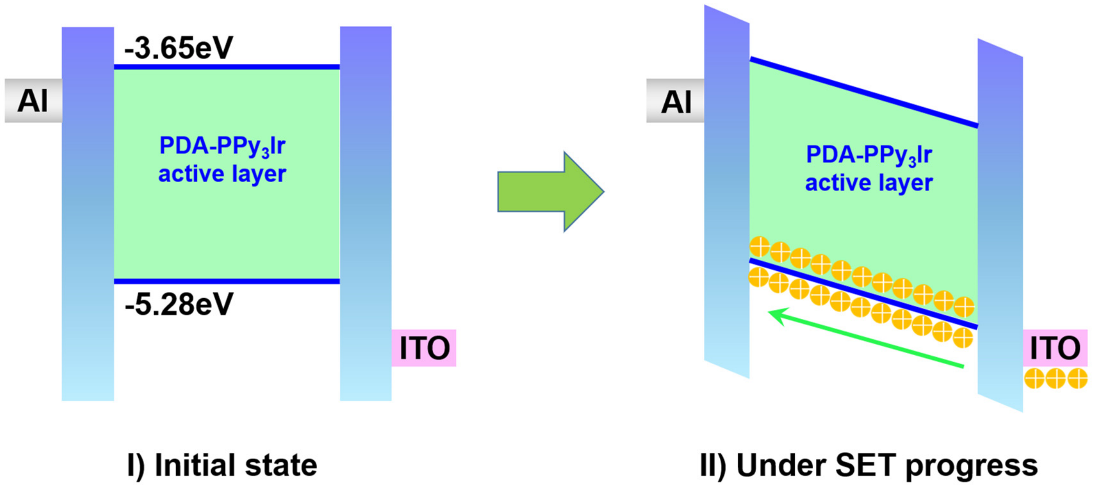

Figure S6. Band diagram of Al/PDA-PPy_3Ir/ITO device.
